# Electrophysiological Features to Aid in the Construction of Predictive Models of Human–Agent Collaboration in Smart Environments

**DOI:** 10.3390/s22176526

**Published:** 2022-08-30

**Authors:** Dor Mizrahi, Inon Zuckerman, Ilan Laufer

**Affiliations:** Department of Industrial Engineering and Management, Ariel University, Ariel 407000, Israel

**Keywords:** coordination, smart environments, mental workload, EEG, Theta/Beta ratio

## Abstract

Achieving successful human–agent collaboration in the context of smart environments requires the modeling of human behavior for predicting people’s decisions. The goal of the current study was to utilize the TBR and the Alpha band as electrophysiological features that will discriminate between different tasks, each associated with a different depth of reasoning. To that end, we monitored the modulations of the TBR and Alpha, while participants were engaged in performing two cognitive tasks: picking and coordination. In the picking condition (low depth of processing), participants were requested to freely choose a single word out of a string of four words. In the coordination condition (high depth of processing), participants were asked to try and select the same word as an unknown partner that was assigned to them. We performed two types of analyses, one that considers the time factor (i.e., observing dynamic changes across trials) and the other that does not. When the temporal factor was not considered, only Beta was sensitive to the difference between picking and coordination. However, when the temporal factor was included, a transition occurred between cognitive effort and fatigue in the middle stage of the experiment. These results highlight the importance of monitoring the electrophysiological indices, as different factors such as fatigue might affect the instantaneous relative weight of intuitive and deliberate modes of reasoning. Thus, monitoring the response of the human–agent across time in human–agent interactions might turn out to be crucial for smooth coordination in the context of human–computer interaction.

## 1. Introduction

In order to design customizable smart environments, it is worthwhile to incorporate the individual traits of the human operator interacting with this environment, so that they would be able to interact and function intuitively across varying scenarios (Smart Home Environment–Agent-Based Models with Scenarios Implementation Support). This task is complicated, since agents might exemplify a wide range of behaviors depending on the context (see a review in [1]). It has already been suggested that achieving successful human–agent collaboration requires the modeling of human behavior for predicting people’s decisions [2,3]. It has been shown, for example, that information about drivers (i.e., driving style) improves the prediction models regarding the use of an automated assistive system [4]. The accuracy of prediction is important, since it can reduce the amount of communication between drivers and automated systems, which will save computational cost. In the same vein, it has been demonstrated that the behavioral economic models of people incorporated into computational approaches enhances the efficacy of advice-provision strategies [5]. Thus, combining behavioral models with computational approaches is important for improving the efficiency of smart environments, since these may assist in smoothing human–agent interactions [6,7].

In recent years, collaborative robots have become major market drivers in Industry 5.0 [8,9,10,11], which aims to incorporate them alongside humans in a wide array of industries and application such as assembly lines, inspection and control of operations [12,13,14], automated advising [15,16], rehabilitation, and search-and-rescue tasks [3]. In collaborative environments involving human–agent teams, sharing of cognitive elements is essential. Thus, the artificial agent is expected to adopt a human-centric strategy, while attempting to perform a collaborative task, and rely on shared goals derived from the human strategy to be effective in assisting the human–agent in performing the joint task [17]. Thus, since Industry 5.0 emphasizes the importance of optimizing human–robot collaboration, having the cognitive reasoning process of the human–agent will make the robot more robust to changes in the environment and more adaptable to different domains. Moreover, emerging mobile technologies are calling for an emphasis on customization of HCI, as the variety of user types is growing and involves aging individuals, young people, and disability concerns. This variety of users calls for more accurate and tailored-made responses from devices than before [18]. To assist in this customization process, EEG can be of much help, since it allows continuous monitoring in a high temporal resolution, utilizing real-time brain signal decoding (RBSD) for gaining insights regarding the current cognitive user state [19].

Previously, we have shown that good coordinators are associated with a higher cognitive load, with respect to weaker coordinators, using the Theta to Beta ratio (TBR) [20]. The goal of the current study was utilizing the TBR and the Alpha band as electrophysiological features that will discriminate between different depth of processing. Specifically, we monitored the modulations of the TBR and Alpha across different task epochs, to model the changes across time, and examined whether the behavior of these electrophysiological markers might differentiate between two cognitive tasks, picking and coordination, which require different depths of reasoning. In the picking condition, participants were requested to freely choose a single word out of a string of four words. In the coordination condition, participants were asked to try and select the same word as the unknown partner that was assigned to them. Participants also underwent a resting-state condition, in which they were required to gaze at a cross situated in the middle of the screen. Hence, the utilization of these two cognitive tasks, picking and coordination, has enabled us to differentiate between two cognitive systems, namely, system 1 and system 2, which correspond to more intuitive and more deliberate reasoning processes, respectively [21,22,23].

To examine the effect of task dynamics, we performed two types of analyses. One that considers the time factor (i.e., observing dynamic changes across trials) and the other that does not. The latter was based on an analysis of the different electrophysiological interactions, by the relative energy level in each EEG band, as a function of the three experimental conditions, without considering the time factor. In the former type, we have also examined the changes in the relative energy at the Alpha frequency band and in the TBR, while considering the temporal changes across trials. Since coordination is a more complex task than picking, it requires a higher level of investment of cognitive resources, and, therefore, we have hypothesized that the Alpha and TBR might behave differently across time in each of the cognitive tasks, picking and coordination.

Results have shown a correspondence between the TBR and the Alpha frequency band across trials, while only in the coordination condition was a clear transition from cognitive load to fatigue observed. Findings have also suggested that there is relationship between the TBR, a marker of cognitive processing, and Alpha power, a marker of arousal [24]. Furthermore, these results demonstrate that, except for the Beta band or the TBR [25], Alpha can also be used as an important feature for constructing a predictive model of human behavior in the context of smart environments.

## 2. Materials and Methods

The main task in the present study was selecting one word out of a string of four different words. The string comprised words from different domains such as cities, drinks, and different car brands. For instance, the following string, Beer, Wine, Water, Whiskey, was used in one of the trials. Overall, there were 12 different strings. In the picking condition, participants were requested to choose a word from the string as they see fit, without further instructions. In the coordination condition, participants were told to choose a word that will match the same word chosen by the unknown player they were randomly assigned to play with. Before the presentation of the strings of words, participants were asked to gaze at a cross situated in the middle of the screen for 2 min (eyes-open condition). The word order within each string remained constant across strings. whereas the order of appearance of the strings was randomized in each condition. There was a three-minute break between each of the three conditions.

Ten participants (right-handed, mean age = 26 years, SD = 4) were enrolled in the study. Upon arrival at the laboratory, they received a verbal explanation, read a written instruction form, and signed an informed consent. Participants were paid according to their level of performance. Specifically, in the picking task, selecting a word rewarded participants with 100 points, otherwise they received nothing. For each successful coordination, participants received 100 points, and in the case of a failure they received nothing. A training session was used prior to the experimental session, in order for the participants to be familiar with the application.

The EEG was recorded by a 16-channel g.USBAMP biosignal amplifier (g.tec, Schiedlberg, Austria), at a sampling frequency of 512 Hz. Sixteen active electrodes were used for collecting EEG signals from the scalp, based on the international 10–20 system. Recording was performed using OpenVibe [26] recording software. Impedance of all electrodes was kept below the threshold of 5K [ohm] during all recording sessions. Data processing was performed using the EEGLAB package [27] in addition to in-house data-processing scripts.

**Figure 1:**
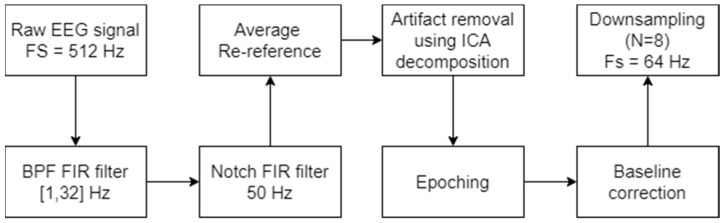
Preprocessing pipeline.

In this study, we have relied on power-spectrum analysis of continuous EEG that reveals the distribution of signal power over different frequency bands (i.e., Delta, Theta, Alpha, and Beta). The data underwent conventional pre-processing stages including filtering, ICA, re-referencing to the average-reference, and down sampling (to 64 Hz) following baseline correction. Analysis was carried on a 1 s epoch window from the onset of each game (see Figure 1 for the preprocessing pipeline). To calculate the coefficients of the four EEG frequency bands for each epoch, we have used the Discrete Wavelet Transform (DWT) [28,29]. To calculate the relative energy, we divided the energy of each band by the sum of all the different bands (for further details regarding EEG analysis, see [20,25,30,31]).

## 3. Results

Statistical analysis was focused only on the frontal and prefrontal electrodes (Fp1, F7, Fp2, F8, F3 and F7), due to the known prefrontal cortex involvement in cognitive processing (e.g., [30,32,33,34,35]). We will first examine the analysis without the time factor and then the analysis with the time factor, while considering the temporal dynamics across the trials.

### 3.1. EEG Frequency Bands’ Interactions as Function of the Experimental Condition

**Figure 2:**
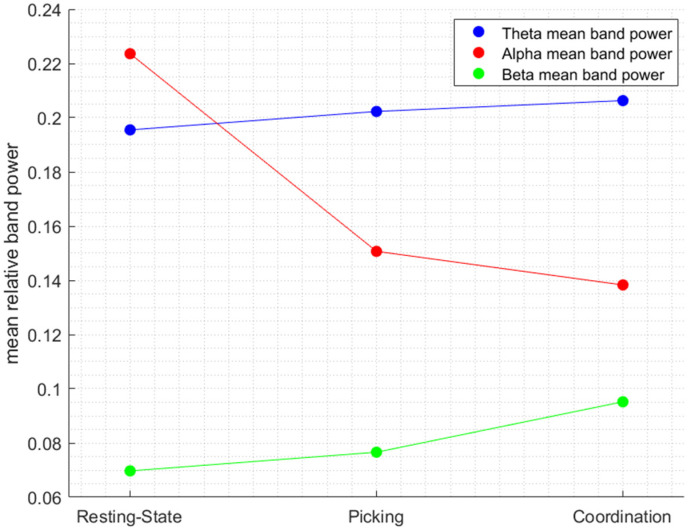
The interaction between experimental state and frequency band.

We have run a two-way repeated-measures ANOVA with the Condition and Frequency bands as independent variables and the relative energy as the dependent variable. The two-way 3 × 3 analysis of variance resulted in a significant Condition × Frequency band interaction (F(4, 15111) = 165.49, *p* < 0.001). Moreover, the main effects of the Condition band (F(2, 15111) = 51.85, *p* < 0.001) and the Frequency band (F(2, 15111) = 5.43, *p* < 0.001) were also significant (see Figure 2).

Figure 2 shows that the intensity of the interaction is different and not uniform for each spectral band. It can be seen that, while, in the Theta band, there is only a small increase in relative power across conditions, in the Alpha and Beta bands, there are pronounced changes. In the Alpha band, there is a large decrease in the relative Beta band, so we can see a larger increase moving from picking to coordination. This power from resting state to picking and a smaller yet noticeable change from picking to coordination. That is, the most salient change for picking to coordination occurs in the Beta band, which corroborates previous findings showing that increase in Beta is associated with enhanced levels of working memory, task engagement, and concentration [36].

To analyze the level of significance in each pair of experimental states for each frequency band, we performed Tukey’s post hoc test [37] in each frequency band. The summary of results is given in Table 1 with classification according to a minimum threshold of *p* < 0.05.

The results in Table 1 shows that the Beta frequency band manages to distinguish between all pairwise comparisons between the conditions, whereas the Alpha frequency band manages to differentiate only between two pairs of contrasts, resting state vs. coordination and resting state vs. picking. In the Theta band, only the contrast between resting state and coordination turned out to be significant. That is, as seen in Figure 2, these findings corroborate the fact that the Beta band is the most sensitive in distinguishing between the two cognitive tasks. In conclusion, it can be seen from the results of our analysis that the higher the frequency band is, the greater the statistical significance of the electrophysiological distinction between all conditions. 

Previous studies have shown that the Alpha frequency band (8–12 Hz) is not only sensitive to mental workload (e.g., [38,39]) but also to reduction in attention or alertness [40]. In addition, there is evidence that an increase in Alpha power is related to lower mental vigilance [40,41] and, hence, a decrease in the attention resources allocated to the task [42]. Despite the above description, the results of the statistical analysis presented in Section 3.1 showed that the relative energy at the Alpha frequency does not constitute an indicator that separates between players who employ a different depth of reasoning (i.e., picking vs. coordination) at a sufficient statistical level (at least *p* < 0.05). One hypothesis, which can explain the lack of statistical significance, is that Alpha power increases over time in tasks that require mental workload (e.g., [43,44]) and that multiple non-stationary processes occur in endogenous Alpha band activity over time [44].

### 3.2. Changes in the Alpha Frequency Band and in the TBR across Trials

In the second type of analysis, which includes the temporal factor, we have performed a comparison of Alpha-frequency intensity as a function of the progression across the experimental trials. Each task in the experiment (picking and coordination) contained 12 questions (see Appendix A) that were assigned into three groups, according to the order of appearance in the experiment: the first stage of the experiment (tasks 1–4), the middle stage (tasks 5–8), and the last stage (tasks 9–12). It should be noted that the sequential order of the questions was randomized and was different for each of the participants.

To examine the effect of the experimental progression, we have run a two-way ANOVA with Stage (first, middle, last) and Condition (coordination, picking) as factors. The results show that there was a significant main effect for Stage, (F(2, 1434) = 80.24, *p* < 0.001), and a significant Stage × Condition interaction, (F(2, 1434) = 67.85, *p* < 0.001).

**Figure 3:**
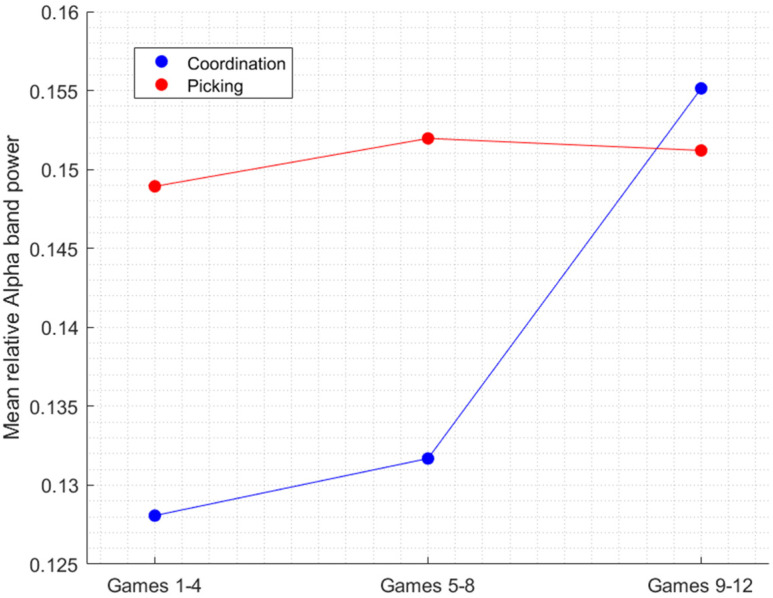
The effect of the interaction between Task and Experimental stage on Alpha.

Figure 3 shows a clear transition visible only in the coordination condition (blue line) from working memory (low Alpha power) to fatigue (increased Alpha power) that occurs in the middle stage of the experiment (games 5–8). The low Alpha power is visible in the first and second stages of the experiment whereas, there is a significant increase in Alpha power in the last stage, until it reaches the level of the Alpha of the picking condition at this stage. In contrast, in the picking condition (red line), Alpha power remained relatively stable across all stages. That is, these findings show that Alpha power can indeed differentiate between picking and coordination, if the temporal aspect of the data is considered.

**Figure 4:**
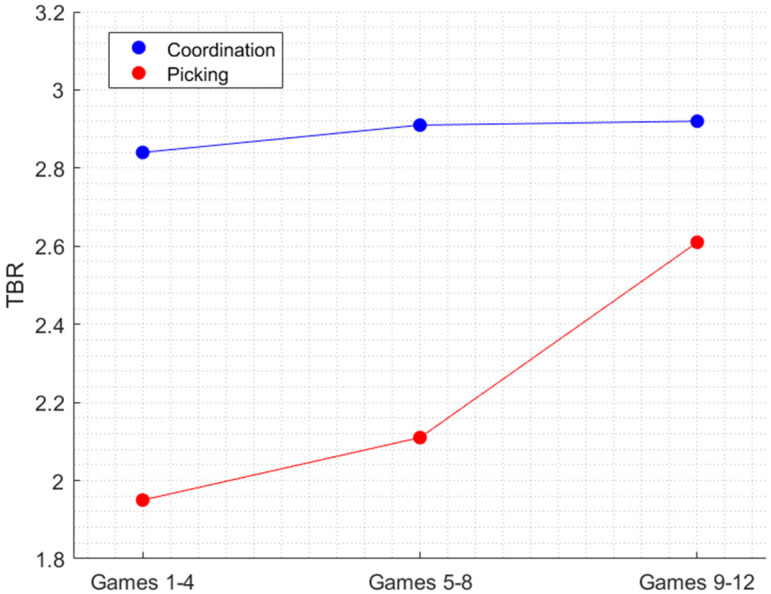
The effect of the interaction between Task and Experimental stage on TBR.

To examine the effect of the temporal progression of the experiment on the Theta and Beta bands, we examined the changes of the TBR index, a measure of cognitive load, across the trials, using the same two-way ANOVA design with Stage and Condition as factors. The clear transition from working memory to fatigue can also be observed in Figure 4 (games 5–8) only in the coordination (blue line). As in the case of the previous analysis, the TBR level associated with the picking condition (red line) remained relatively constant across the experimental stages, whereas in the coordination condition there is an overall sharp increase in the TBR as a function of the experimental stages. Moreover, the slope between the middle and late stages of the experiment is higher than in earlier stages. Note that as the level of the TBR increases, the level of cognitive load decreases. Taken together, these results demonstrate that there is relationship between the TBR, a marker of cognitive processing, and Alpha power, a marker of arousal.

## 4. Discussion

The motivation for the current study stems from the assumption that, in the context of smart environments, it is important to monitor modulations of the human response across time to more precisely model human behavior. In this study, the goal was to utilize the TBR and the Alpha band as electrophysiological markers that will enable differentiation between two cognitive tasks, picking and coordination, each requiring a different depth of reasoning. Since coordination is a more complex task than picking, and it requires a higher level of investment of cognitive resources, we expected that the Alpha and TBR will demonstrate differential activation across time as a function of the cognitive condition (picking, coordination). When the temporal factor was not considered, only Beta was sensitive to the difference between picking and coordination. However, when the temporal factor was included, a clear transition point, evident only in the coordination condition, was clearly visible between cognitive effort and fatigue in the middle stage of the experiment (stages 5–8). This transition point was evident either when the electrophysiological measure was relative to the Alpha power or TBR. Since the Alpha band could not significantly differentiate between picking and coordination when the temporal factor was not considered, we repeated the analysis, implicating Alpha when the task was divided into time bins. These results highlight the importance of monitoring the electrophysiological indices across time, as different factors such as fatigue might affect the instantaneous relative weight of intuitive and deliberate modes of reasoning (e.g., [45,46]). Thus, monitoring the response of the human–agent across time in human–agent interactions might turn out to be crucial for smooth coordination in the context of human–computer interaction.

Furthermore, the current findings indicate that there is a relationship between Alpha, a marker of cognitive processing, and the TBR, a marker of cognitive load. In the current literature, there are mixed results regarding the relationship between the Theta/Beta ration and the Alpha frequency band. Previous research has indicated that the Theta/Beta ratio is not related to the Alpha frequency band as an index of arousal [24]. In contrast, in another piece of research, the Theta and Alpha frequency bands showed similar trends in synchronization across different cognitive tasks, while Beta has shown the opposite trend [39]. However, our results show a possible connection between the Theta/Beta ratio and Alpha, as an index of arousal. Specifically, the Theta/Beta ratio and Alpha showed a very similar trend along task progression, and the transition from task engagement to disengagement occurred at a similar time point.

The findings of our study carry practical implications. Overall, our findings can contribute to the development of agent models dealing with human–agent interaction, where collaboration is constrained by the cost of communication. The electrophysiological features extracted in our study, either the Alpha or TBR, could be incorporated into an agent model, to assist in deciding when it is worthwhile to communicate, while taking into consideration the associated cost. For example, a smart centralized air-conditioning system would intend to accurately and tacitly predict the most convenient ambient temperature for the human–agent, while minimizing trial and error interactions. In scenarios like these, the intelligent agent needs to trade off the cost of communication against its potential benefits [7,47].

Our features can also aid in reinforcement learning (e.g., [48]), where the intelligent agent needs to attribute a value to a certain state. When multi-agents are involved, this task is more complicated and, therefore, the agent can use opponent modeling to estimate the policies employed by other agents and compute the expected probabilities of the joint actions of the other agents [49]. The finding that the Alpha and TBR might replace each other as possible features in predictive models carries practical implications. There is a growing commercial market for single-electrode portable EEG devices (e.g., [50,51]). Therefore, measuring the Alpha band becomes highly feasible. Commercial systems might not only easily record the Alpha band, without the need for any additional computations, but also utilize it in real-time to customize the environment to fit human preferences.

There are a number of possible directions for future research. It would be worthwhile constructing an agent that will be able to construct a predictive model based on additional electrophysiological features, except for the Alpha and TBR. Such models could also combine electrophysiological and behavioral measures, such as social value orientation (SVO) [52,53]. Furthermore, behavioral experiments have shown that the behavior of players in coordination games is influenced by a variety of factors such as loss aversion [54], social value orientation [55,56], revenues distribution [55], and culture [56,57]. Therefore, it will be interesting to incorporate the relevant electrophysiological correlates of these different factors as features in the agent predictive models.

## 5. Conclusions

Our findings highlight the importance of monitoring the electrophysiological indices, as different factors such as fatigue might affect the instantaneous relative weight of intuitive and deliberate modes of reasoning. Thus, monitoring the response of the human–agent across time in human–agent interactions might turn out to be crucial for smooth coordination in the context of human–computer interaction. Moreover, the Theta/Beta ratio and Alpha showed a very similar trend along task progression, and the transition from task engagement to disengagement occurred at a similar time point. Thus, these findings suggest that there is relationship between the TBR, a marker of cognitive processing, and Alpha power, a marker of arousal [24].

## Figures and Tables

**Table 1:** Tukey’s post hoc test results summary, according to division into frequency bands.

Picking–Coordination	Resting State–Picking	Resting State–Coordination	
✗	✗	✓	**Theta**
*p* = 0.7675	*p* = 0.2561	*p* < 0.05	**[4,8] (Hz)**
✗	✓	✓	**Alpha**
*p* = 0.1095	*p* < 0.001	*p* < 0.001	**[8,16] (Hz)**
✓	✓	✓	**Beta**
*p* < 0.001	*p* < 0.001	*p* < 0.001	**[16,32] (Hz)**

## Data Availability

The data presented in this study are available on request from the corresponding author. The data are not publicly available due to participants privacy.

## Appendix A. Tacit Coordination Game List

In this appendix, we will describe the set of tacit coordination games, which includes 12 games, that were designed in order to evaluate the individual coordination abilities of the various players, together with their electrophysiological patterns in different cognitive hierarchy levels. The full game list is presented in Table A1. It should be noted that the words on the game boards appeared in Hebrew, which is the native language of the participants.

**Table A1:** Experimental game list.

Game Number	Option 1	Option 2	Option 3	Option 4
**1**	Water	Beer	Wine	Whisky
**2**	Tennis	Volleyball	Football	Chess
**3**	Blue	Gray	Green	Red
**4**	Iron	Steel	Plastic	Bronze
**5**	Ford	Ferrari	Jaguar	Porsche
**6**	1	8	5	16
**7**	Haifa	Tel-Aviv	Jerusalem	Netanya
**8**	Spinach	Carrot	Lettuce	Pear
**9**	London	Paris	Rome	Madrid
**10**	Hazel	Cashew	Almond	Peanut
**11**	Strawberry	Melon	Banana	Mango
**12**	Noodles	Pizza	Hamburger	Sushi

The position of the questions appearing on the game screen is fixed and follows the order of the questions shown in Table A1. This decision in the design of the experiment was made to create a uniform experimental set-up between the various actors and to neutralize the possible effect of spatial cues.

## Appendix B. Training Tasks Game List

This appendix presents the training tasks, which were transferred between stages 2 and 3 of the experiment. The purpose of these tasks is to verify the players’ technical understanding of the application, before performing the actual experiment. From a review of Table A2, it can be seen that there is no overlap in the content of the training tasks with the experiment tasks.

**Table A2:** Training game list.

Game Number	Option 1	Option 2	Option 3	Option 4
**1**	Sapphire	Glass	Emerald	Diamond
**2**	Lion	Panther	Frog	Tiger
**3**	Boat	Helicopter	Bicycle	Plane
**4**	Thursday	Tuesday	Saturday	Sunday
**5**	2019	2000	1995	1997
